# Association among Autistic Traits, Treatment Intensity and Outcomes in Adolescents with Anorexia Nervosa: Preliminary Results

**DOI:** 10.3390/jcm10163605

**Published:** 2021-08-16

**Authors:** Jacopo Pruccoli, Simone Rosa, Carlo Alberto Cesaroni, Elisabetta Malaspina, Antonia Parmeggiani

**Affiliations:** 1 IRCCS Istituto delle Scienze Neurologiche di Bologna, Centro Regionale per i Disturbi della Nutrizione e dell’Alimentazione in Età Evolutiva, U.O. di Neuropsichiatria dell’Età Pediatrica, 40138 Bologna, Italy; jacopo.pruccoli@studio.unibo.it (J.P.); simone.rosa5@studio.unibo.it (S.R.); carlo.cesaroni@studio.unibo.it (C.A.C.); elisabetta.malaspina@aosp.bo.it (E.M.); 2 Dipartimento di Scienze Mediche e Chirurgiche (DIMEC), Università di Bologna, 40138 Bologna, Italy

**Keywords:** autism spectrum disorder, outcome, need for treatment, anorexia nervosa, ADOS-2, autism-spectrum quotient, psychopharmacologic treatment

## Abstract

The present study investigates the impact of Autism Spectrum Disorder (ASD) traits on the treatment intensity and outcomes (psychopathology and weight) of 22 adolescent inpatients with Anorexia Nervosa (AN), who were selected on the basis of suspected ASD traits. ASD traits were measured at admission (T0) using the Autism Diagnostic Observation Schedule-Second Edition (ADOS-2) and the Autism-Spectrum Quotient (AQ). Psychopathology was measured with Eating Disorder Inventory-3 (EDI-3) and Self-Administered Psychiatric Scales for Children and Adolescents (SAFA) at admission and discharge (T0, T1). Percentage BMI was assessed at admission, discharge, first follow-up (T2, 7–22 days) and second follow-up (T3, 22–45 days). Results were controlled for age and EDI-3 global psychological maladjustment. When compared with other patients with AN, AN individuals with ADOS-2 and AQ diagnostic scores for ASD showed overlapping types of treatments, as well as psychopathological and weight outcomes. ASD total scores were not correlated with treatment intensity or treatment outcomes. Preliminary results show that ASD traits do not impact treatment intensity and outcomes in adolescents with AN and suspected ASD traits.

## 1. Introduction

Anorexia Nervosa (AN) is an eating disorder (ED) characterized by a significantly low body weight due to restricted food intake, an intense fear of gaining weight, and a disturbance in self-perceived body image (American Psychiatric Association 2013) [1]. AN is more common in females [2], the onset of the disorder is usually during adolescence and its lifetime course is highly variable. Autism Spectrum Disorder (ASD) is characterized by a behavioural triad of impairments: in social reciprocity, social communication, and flexibility of thought and behaviour [1]. These difficulties are thought to be dimensional, with autism representing the extreme of highly heritable traits that can be documented in different clinical populations [3]. Symptoms of ASD may show extreme clinical variability leading to different degrees of impairments in daily life [4]. A widely used measure of autistic traits is the Autism Diagnostic Observation Schedule (ADOS) [5]. ADOS is a semistructured, standardized assessment of social interaction, communication, play, and imaginative use of materials for individuals suspected to have ASD. Although their incidence is growing, both ASD and AN are uncommon disorders in the general population. However, ASD appears to be over-represented within eating-disorder populations [6], with a recent systematic review reporting a mean prevalence of 22.9% [7]. Much of the research on ASD in AN comes from a Swedish cohort, members of which were assessed for ASD at regular intervals over a 16-year period [8]. In each of the four assessments, the diagnostic tools used to evaluate ASD were different, yielding differing rates of ASD diagnosis. This underlines the difficulty of assessing ASD in AN, particularly in light of evolving diagnostic criteria and the presence of a distinct female autism phenotype [9], which makes it challenging to accurately gauge the disorder in females [10]. A recent review by Westwood and Tchanturia [11] assessed the presence of ASD traits in adolescents with AN and documented a variable prevalence (10–52.5%). It is also possible that the acute, starved state associated with AN exacerbates the presence of symptoms characteristic of ASD [12]. This state versus trait argument [12] calls for further research to disentangle the complex relationship between AN and ASD. It is important to highlight the distinction between interventions for ASDs and interventions for EDs, as they serve vastly different purposes [13]. Interventions for aspects of autism typically involve a range of therapeutic strategies aimed to reduce the functional impact of autistic traits on the daily life of the affected individual and improve long-term outcomes. On the other hand, EDs, and specifically AN, have the highest mortality rate of any psychiatric condition [14]. ED treatments, therefore, are aimed at patients’ recovery by restoring healthy body weight after starvation and by targeting socio-emotional factors that are believed to contribute to the persistence of the disorder [15]. For some groups of patients with EDs, such as young patients with AN, family-based treatment (FBT) represents the main treatment options. Even if there is no indication for using specific psychotropic drugs to address core symptoms of AN [16], in severe conditions and comorbidities drug therapy may represent an important option. In recent years such antidepressants as selective serotonin reuptake inhibitors (SSRI) and antipsychotic (AP) drugs, all second-generation antipsychotics (SGA), have been used to manage a number of psychiatric disorders in children and adolescents. These psychotropic drugs are not officially approved for any ED in this age group, at least as the only treatment of choice [17]. Nevertheless, their use has significantly increased, and nowadays many clinicians prescribe them for symptomatic treatment of comorbid conditions in AN [18,19].

It is questionable if autistic traits in adolescent patients with AN could influence the treatment and prognosis of ED. For this reason, we investigated this question in our previously reported sample [6] to evaluate if AN patients with higher levels of autistic traits require different and more intensive treatments and reach poorer outcomes than AN patients with lower levels of autistic traits.

## 2. Materials and Methods

### 2.1. Design

The patients were enrolled in this study from 1 January 2015 to 31 December 2019 and came from a population of adolescents in the care of the Italian Regional Centre of Feeding and Eating Disorder of patients of developmental age in Bologna, Italy. Inclusion criteria were a diagnosis of AN and an age between 11 and 17 years at the time of hospitalization, as well as the presence of clinical psychopathological traits for ASD. Patients who were unable to attend a complete clinical evaluation, as shown below, and who did not have proper follow-up documentation, were excluded from the study. Patients whose initial assessment revealed that AN was due to a concurrent psychotic, bipolar disorder, or neurological condition, were also excluded. The diagnosis of AN was performed by child neuropsychiatrists and psychologists with experience in ASD and ED. All patients were treated in accordance with psycho-nutritional guidelines, particularly with Family Therapy for Anorexia Nervosa (FT-AN) [17]. Inclusion and exclusion criteria did not limit the enrolment on the basis of gender, and males were also considered for this study in order to document a complete follow-up for male individuals with AN due to the scarce evidence available in literature for this specific sub-population. Some patients concurrently received psychopharmacologic treatment, which was tailored to their specific clinical and psychopathological condition. The decision to administer a specific antidepressant and/or antipsychotic treatment was taken on the basis of routine clinical management for patients with AN according to international clinical guidelines [17], and independently of the enrolment in this study.

### 2.2. Measures

This study investigated demographic measures, autistic traits, psychopathology, adopted treatments, and clinical outcomes. Demographic data included age, gender, presence of a familial history of EDs or ASD, and past experience of traumatic events. Diagnoses of AN were performed according to DSM-5 criteria [1] on the basis of clinical judgment and were supported by the results of the EDI-3 and SAFA questionnaires. The possible presence of traits suggestive for ASD was assessed by a child neuropsychiatrist and a clinical psychologist following their clinical judgement. The examiners searched for the following signs during the psychiatric examination performed at admission, and during the anamnestic interview with the caregivers: deficits in social-emotional reciprocity; deficits in nonverbal communicative behaviors; deficits in developing, maintaining, and understanding relationships; stereotyped or repetitive motor movements, use of objects, or speech; insistence on sameness; inflexible adherence to routines, or ritualized patterns of behavior; restricted, fixated interests and hyper or hyporeactivity to sensory input. Patients with at least one of these features, as revealed by clinical examination and/or anamnestic interview, were considered for inclusion in the study. Once the patients were enrolled in the study, ASD traits were investigated by administering the ADOS-2 protocol [5] and the AQ questionnaire [20,21]. The ADOS-2 is a standardized test for the assessment of existing behaviors indicative of ASD [22]. ADOS-2 Module 4, which is used to assess adolescents and adults without language impairment, presents a score in the domains of language and communication, reciprocal social interaction, imagination, creativity, stereotyped behavior, and restricted interests. The AQ test explores different domains such as social skills, attention shifting, attention to detail, communication, and imagination.

Psychopathology was investigated by administering the EDI-3 questionnaire [23] and the Self-Administered Psychiatric Scales for Children and Adolescents (SAFA) test [24]. EDI-3 assessed ED pathological traits, both at hospital admission and at hospital discharge. SAFA represents a unitary instrument that preliminarily assesses psychiatric conditions and reports different psychopathological scales. Its use has been tested and is indicated for screen psychiatric comorbidities in young patients with ED [25]. For the purpose of this study, patients were screened for comorbidity with major depressive disorder and/or obsessive-compulsive disorder (OCD) according to specific SAFA subscales (SAFA-D, depression and SAFA-O, OCD symptoms, respectively) and clinical impression following DSM-5 criteria [1]. 

Adopted treatments were investigated in order to document whether patients with ASD comorbidity needed more intensive treatment options. To this end, number and duration of hospital admissions, possible pediatric ward admissions, psychopharmacological treatments with antidepressants, antipsychotics and mood stabilizers, and relative dosage in mg, were considered. Treatment intensity was evaluated considering the following elements: number of hospital admissions, number of admissions in a Pediatric Emergency Unit (PEU), duration of hospitalization, antidepressants (yes/no) (sertraline, fluoxetine, fluvoxamine) and antipsychotics (yes/no) (risperidone, aripiprazole, olanzapine) used. The number of hospitalizations was assessed as the total number of hospital admissions before being enrolled in the study. These included all types of hospital admissions due to AN, apart from PEU admissions, which represented a separate variable. Italian local health authority mandates that children and adolescents with severe eating disorders and in need of specific inpatient treatment be admitted to child and adolescent neurology and psychiatry units or general pediatric departments.

In order to investigate treatment outcomes, modifications in body weight and psychopathology were assessed at multiple times during the course of treatment. For the purpose of this study, for each patient we prospectively collected data related to the clinical outcome at the start of hospitalization (T0), upon discharge from our ward (T1), at the first scheduled post discharge evaluation (T2, between 7 and 21 days from discharge) and at the last post discharge evaluation (T3, between 22 and 45 days from discharge).

Body weight and its modifications were assessed as %BMI. The use of this measure is indicated by the report Junior MARSIPAN: Management of Really Sick Patients under 18 with Anorexia Nervosa [25]. Percentage BMI is calculated as (BMI/median BMI for age and gender × 100) [26]. The World Health Organization BMI-for-age charts for girls and boys were used as reference values in this study [27]. BMI and body weight were recorded as well. Modification in ED psychopathology was assessed as changes in EDI-3 ED-specific (EDRC, Eating Concerns) and non-ED-specific (GPMC, Global Psychological Maladjustment) subscales. Modifications in depressive and OCD-psychopathology were assessed as changes in SAFA-D (Depression) and SAFA-O (OCD symptoms) subscales. The full timeline for outcome assessment in the study is reported in Table 1.

Treatment intensity and psychopathological outcomes were screened for possible associations with ASD measures. This screening was conducted at a diagnostic level (ADOS-2/AQ scores compatible with a diagnosis of ASD), at a total score level (ADOS-2/AQ total scores), then the variation of %BMI over time (weight outcome) was screened for possible associations with ADOS-2/AQ scores compatible with a diagnosis of ASD.

### 2.3. Ethical Considerations

The study was approved by the Ethical Committee (protocol code: ASD-AN-18). Written informed consent was obtained.

### 2.4. Statistical Analysis

Descriptive analyses of demographics, treatment and outcome measures, and total scores and subscales of ADOS-2, AQ, EDI-3 and SAFA were performed. The Shapiro-Wilk test was used to assess normality of data distribution. Student’s T-tests were used to assess possible differences in continuous variables between groups of patients (Mann-Whitney for non-normally distributed variables). Pearson’s r (Spearman’s rho for non-normally distributed variables) correlations were calculated between continuous variables. Bonferroni correction was applied for multiple comparisons. Stepwise multiple logistic regressions were conducted to assess possible differences between patients with and without ASD diagnostic scores regarding treatment intensity and psychopathological outcomes (T0–T1). Stepwise multiple linear regressions were conducted to assess possible correlations between ASD total scores and treatment intensity or psychopathological outcomes. Then, differences were assessed between patients with and without diagnostic ASD scores concerning %BMI improvement across repeated measurements (T0, T1, T2, T3). This assessment was carried out with repeated measures ANOVA, with time as a repeated measure factor (T0, T1, T2, T3) and an ADOS-2 or AQ diagnostic score as a between factor variable. All analyses were controlled for baseline global psychological maladjustment as measured by EDI-3 (GMPC) and age. Statistical analyses were conducted using JASP, version 0.14.1 for Windows. Adopted alpha error rate was 0.05 (two-tailed), with a conservative statistical power of 95%. Descriptive statistics for demographic and clinical variables were calculated.

## 3. Results

### 3.1. Demographics

During the period of the study, 82 patients (78 females, 4 males) with AN referred to our centre, with a mean age of 14.8 years. The mean BMI at admission was 14.2 kg/m^2^, with a mean duration of untreated illness of 14.0 months. Twenty-three patients met the inclusion criteria. Due to insufficient clinical documentation, one patient was lost to follow-up. Thus, twenty-two patients were enrolled in this study (20 females and 2 males). All patients received a diagnosis of AN; 15 (48.4%) of them had comorbid OCD, while 8 (25.8%) presented symptoms compatible with a comorbid major depressive disorder. The mean age at admission was 14.6 (±1.3) years. The mean duration of hospitalization was 3.8 (±2.5) months. Three patients (9.7%) had a family history of EDs, but no family member with ASD was identified. Table 2 reports the comparisons between demographic and clinical variables at the moment of admission of patients with ADOS-2 + v. ADOS-2− scores, and with AQ + v. AQ − scores. No significant difference emerged at baseline for demographic and clinical variables between these groups of patients.

### 3.2. ASD Traits and Psychopathology

All patients completed selected tests (ADOS-2, AQ, EDI-3, SAFA). Notably, four of 22 (18.2%) patients obtained ADOS-2 scores consistent with a diagnosis of ASD. The mean total score at ADOS-2 was 4.1 (±5.1). Five patients out of 22 (22.7%) obtained AQ scores compatible with ASD, and the mean total score was 21.6 (±7.9). Neither ADOS-2 (*p* = 0.194) nor AQ scores (*p* = 0.671) were significantly different in patients with a major depressive disorder. Neither ADOS-2 (*p* = 0.249) nor AQ scores (*p* = 0.434) were significantly different in patients with OCD.

### 3.3. Psychopharmacological Treatments

All patients received at least one psychopharmacological treatment. Sixteen (72.7%) patients received antipsychotics, and 21 (95.5%) patients received selective serotonin reuptake inhibitors (SSRI). As for antipsychotics, five (22.7%) patients were treated with risperidone at a mean dosage of 1.3 (±1.0) mg, six (27.3%) patients with aripiprazole at a mean dosage of 7.8 (±4.0) mg, and seven (31.8%) patients with olanzapine at a mean dosage of 8.6 (±5.2) mg. As for SSRI, 17 (77.3%) patients were treated with sertraline (mean dosage 72.1 ± 34.1 mg), eight (36.4%) patients with fluvoxamine (mean dosage 109.4 ± 82.3 mg), and three (13.6%) patients with fluoxetine (mean dosage 30.0 ± 10.0 mg). One patient received aripiprazole and olanzapine concurrently.

### 3.4. ASD Traits and Treatment Intensity

Treatment intensity was not different in patients with and without ASD diagnostic scores. The comparisons between drug treatment variables of patients with ADOS-2 + v. ADOS-2− scores, and AQ + v. AQ− scores, are reported in Table 3. The comparisons between these groups of patients concerning the number of received hospital interventions are reported in Table S1.

Treatment intensity was not correlated with ASD total scores at ADOS-2 or AQ. Correlations between these variables and the number of received hospital interventions are reported in Table S1. Correlations concerning drug treatments are reported in Table S2.

### 3.5. Clinical Outcomes

The mean %BMI was 71% (±8.9) at admission (T0) and 79.7 (±7.4) at discharge (T1). Between admission and discharge (T1–T0), the main %BMI difference was +8.3% (±5.3). Between discharge and the first follow-up (T2–T1), the main %BMI difference was +1.0% (±2.5). Between discharge and the second follow-up (T3–T1), the main %BMI difference was +3.4% (±4.8). Concerning psychopathological outcomes, the following improvements were registered: EDI-3 EDRC: −6.6 (±29.4); EDI-3 GMPC: −14.7 (±21.3); SAFA-D: −12.2 (±12.8); SAFA-O: −6.0 (±8.9).

### 3.6. Clinical Outcome in Patients with and without ASD

Psychopathological outcomes were not different in patients with and without ASD diagnostic tests. The improvement in psychopathological outcomes was not correlated with ASD total scores. Correlations between ASD tests and psychopathological outcomes are reported in Table 4.

With regard to differences in %BMI over time, ADOS-2 and AQ diagnostic scores did not show significant results concerning differences in clinical improvement. No significant between-subjects association resulted (F (1,11) = 0.069, *p* = 0.799; η2 = 0.006). No significant between-subjects association resulted between AQ diagnostic scores (positive/negative) and %BMI values over time (F (1,11) = 1.612, *p* = 0.236; η2 = 0.104).

## 4. Discussion

The purpose of this study was to investigate the impact of ASD traits on treatment intensity and outcomes in a group of adolescents hospitalized with AN. Our initial hypothesis was that patients with higher ASD traits would need more intensive psychopharmacological treatments and hospital interventions.

Recent guidelines accept inpatient treatment for cases with AN and severe metabolic impairment, but less intensive treatment environments should be adopted when possible [16]. We found no significant difference in number of hospital admissions, number of PEU admissions, and duration of hospitalization between patients with high AQ scores and patients with low AQ scores. Therefore, the presence of a higher level of ASD traits in patients with AN does not appear to predispose them to the risk of a higher number of hospitalizations and PEU admissions. This finding partially contradicts the results of Tchanturia and colleagues [28]. In that study, the authors found that ASD-traits may impact the treatment outcome of cognitive symptoms of AN. These conflicting results lead us to hypothesize that the negative impact of ASD traits may affect the cognitive rehabilitation of AN, but not necessarily lead to more intensive drugs or hospital treatments.

Regarding the intensity of received treatment, we found no significant difference between patients with and without ASD diagnostic scores, and also with ASD total scores. These results are in contradiction with our initial hypothesis. Antidepressant and antipsychotic should not be offered as the sole treatments for AN [17], unless atypical antipsychotics may be of some benefit in carefully selected clinical settings [16]. Relevantly, there are no drugs approved for treating core symptoms of ASD, even though psychopharmacological interventions could improve ASD-associated symptoms that adversely impact the life of individuals with ASD [29].

As for treatment outcomes (improvement in psychopathological and weight variables), we found no significant difference between patients with and without ASD diagnostic scores. We also did not find a correlation between total ASD scores and treatment outcomes. These results partially conflict with previous studies documenting that individuals with high ASD traits may show reduced response to cognitive remediation therapy (CRT) [28]. Other authors, however, found no difference in Morgan Russell Outcomes at discharge for AN individuals with high and low ASD traits [30]. Thus, we hypothesize that the negative contribution of ASD traits to the treatment of AN should be categorized by further studies, which should investigate the response of AN individuals with ASD traits to different treatment modalities.

Our study considered a sample that has been previously described [6] to perform a prospective assessment of a subset of young patients with AN and suspected ASD traits, systematically tested. The study had some limitations: (a) a small sample size, although our patients were systematically tested for a series of variables in a standardized, clinician-guided assessment providing a thorough clinical description of a selected population; (b) there was no control group, since this study specifically aimed at a prospective assessment in selected patients with a possible presence of ASD traits; (c) measurements of ASD traits were obtained only once during the follow-up, and further studies should consider administering standardized ASD measures at repeated intervals to assess possible effects of treatments for ED on ASD traits; (d) since the patients were enrolled on the basis of the presence of suspected clinical traits suggestive for ASD, the results of this study should not be generalized to clinical populations with different inclusion criteria; (e) the presence of two males among the selected 22 cases could have unbalanced the total sample, since males with AN may represent specific features in terms of clinical presentations and outcomes [31].

Our study also had significant strengths. First, different from previous research [30], the evaluation for both ASD and ED psychopathology was performed in a clinical setting by child neuropsychiatrists and psychologists trained in the field of ASD and ED. Second, outcome measures included differences in body weight (%BMI), which permitted us to evaluate the impact of ASD traits on different treatment goals for ED, distinguishing the contributions of ED psychopathology, body weight and ASD traits in the treatment of AN. The evidence garnered in this study with regard to the different need for treatment of AN patients with ASD traits may encourage new research on the treatment of ED.

## 5. Conclusions

Based on these preliminary results, AN individuals with ADOS-2 or AQ diagnostic scores for ASD did not receive different psychopharmacological treatments or hospitalizations, nor obtained different psychopathological or weight treatment outcomes when compared with other patients having AN. ASD total scores were not correlated with treatment intensity or treatment outcomes. These results should be assessed in light of previous studies documenting a negative effect of ASD traits on cognitive treatments for AN. However, it is reasonable to think that other factors may influence the psychological, psychopharmacological, and nutritional treatment of AN. An early detection of those elements may lead to more proper clinical care. 

## Figures and Tables

**Table 1:** Timeline of the study.

T	Timing	Outcomes Assessed
T0	Admission	%BMI, EDI-3 (EDRC, GMPC) SAFA-D, SAFA-O
T1	Discharge	%BMI, EDI-3 (EDRC, GMPC) SAFA-D, SAFA-O
T2	First follow-up (7–21 days)	%BMI
T3	Second follow-up (22–45 days)	%BMI

Abbreviations: AQ = Autism Questionnaire; ADOS-2 = Autism Diagnostic Observation Schedule-2; EDI-3 = The Eating Disorder Inventory-3; EDRC = Eating Concerns; GPMC = Global Psychological Maladjustment; SAFA-D = Self-Administered Psychiatric Scales for Children and Adolescents, Depression scale; SAFA-O = Self-Administered Psychiatric Scales for Children and Adolescents, Obsessive-compulsive symptoms; T0: at the start of hospitalization; T1: upon discharge from our ward; T2: at the first scheduled post discharge evaluation; T3: at the last post discharge evaluation.

**Table 2:** Comparisons of clinical variables at admission between ASD+ and ASD− patients with AN.

	ADOS-2			AQ		
	ADOS-2+	ADOS-2−	*p*-Value	AQ+	AQ−	*p*-Value
Variables						
Age	15.9 ± 1.1	15.8 ± 2.8	0.859	15.7 ± 1.4	16.6	0.389
Gender	17 F (94.4%) 1 M (5.6%)	3 F (75.0%) 1 M (25.0%)	0.267	16 F (94.1%) 1 M (5.9%)	4 F (80.0%) 1 M (20.0%)	0.387
Clinical variables at admission						
%BMI	71.1 ± 9.0	72.7 ± 9.3	0.774	70.3 ± 8.4	75.3 ± 10.1	0.359
EDI-3 EDRC	60.6 ± 20.6	72.0 ± 27.9	0.327	60.4 ± 23.6	70.2 ± 13.3	0.388
EDI-3 GMPC	63.5 ± 28.5	81.5 ± 16.5	0.268	60.2 ± 27.6	89.2 ±6.9	0.019
SAFA-D	63.3 ± 14.4	75.5 ± 13.8	0.253	63.7 ± 15.5	74.0 ± 8.5	0.118
SAFA-O	52.9 ± 11.5	57.5 ± 9.5	0.538	51.4 ± 10.1	63.3 ± 10.9	0.234

Abbreviations: ASD = Autism Spectrum Disorder; AQ = Autism Questionnaire; ADOS-2 = Autism Diagnostic Observation Schedule-2; EDI-3 = The Eating Disorder Inventory-3; EDRC = Eating Concerns; GPMC = Global Psychological Maladjustment; SAFA-D = Self-Administered Psychiatric Scales for Children and Adolescents, Depression scale; SAFA-O = Self-Administered Psychiatric Scales for Children and Adolescents, Obsessive-compulsive symptoms. Note: Bonferroni-corrected significance level for EDI-3 and SAFA scores = 0.05/4 = 0.013. Statistically significant differences marked in bold.

**Table 3:** Comparisons of psychopharmacological treatments between ASD+ and ASD- patients.

	ASD Measures					
	ADOS-2+			AQ+		
	OR	C.I.	*p*-Value	OR	C.I.	*p*-Value
Risperidone	4.262^−8^	−7256–−7222	0.996	2.340^−8^	−5710–5675	0.995
Aripiprazole	19.877	−0.504–6.483	0.094	0.611	−3.328–2.342	0.733
Olanzapine	1.569	−3.662–4.563	0.830	0.022	−8.511–0.858	0.109
Sertraline	0.530	−3.755–2.484	0.690	0.213	−4.647–1.556	0.329
Fluvoxamine	0.851	−3.069–2.746	0.913	0.235	−4.216–1.322	0.306
Fluoxetine	3.943	−2.014–4.758	0.427	179.787	−2.186–12.569	0.168

Abbreviations: AQ = Autism Questionnaire; ADOS-2 = Autism Diagnostic Observation Schedule-2; ASD = Autism Spectrum Disorder. Note: results controlled for baseline EDI-3 Global Psychological Maladjustment (GMPC) and age.

**Table 4:** ASD traits and psychopathological outcomes.

	ASD Measures											
	ADOS-2 Total Scores			AQ Total Scores			ADOS-2 Diagnostic Scores for ASD			AQ Diagnostic Scores for ASD		
Treatments	F	*p*-Value	Adjusted R2	F	*p*-Value	Adjusted R2	F	*p*-Value	Adjusted R2	F	*p-*Value	Adjusted R2
EDI-3 EDRC	1.514	0.714	0.075	1.502	0.743	0.073	1.498	0.752	0.073	1.892	0.325	0.123
EDI-3 GMPC	2.185	0.702	0.158	2.153	0.775	0.154	2.133	0.841	0.152	2.615	0.315	0.203
SAFA-D	0.275	0.842	−0.147	0.343	0.604	−0.131	0.295	0.709	−0.142	0.252	0.882	−0.152
SAFA-O	0.281	0.596	−0.145	0.188	0.879	−0.167	0.765	0.453	−0.122	0.472	0.373	−0.103

Abbreviations: AQ = Autism Questionnaire; ADOS-2 = Autism Diagnostic Observation Schedule-2; EDI-3 = The Eating Disorder Inventory-3; EDRC = Eating Concerns; GPMC = Global Psychological Maladjustment; SAFA-D = Self-Administered Psychiatric Scales for Children and Adolescents, Depression scale; SAFA-O = Self-Administered Psychiatric Scales for Children and Adolescents, Obsessive-compulsive symptoms. Note: results controlled for baseline EDI-3 GMPC and age.

## Data Availability

The data sets used and analyzed during the current study are available from the corresponding author on reasonable request.

## Supplementary Materials

The following are available online at https://www.mdpi.com/article/10.3390/jcm10163605/s1, Table S1: Comparisons of number and length of hospital admissions between patients with and without clinical scores compatible with a diagnosis of ASD; Table S2: ASD total scores and treatment intensity.
