# Supplementary material for: Association among Autistic Traits, Treatment Intensity and Outcomes in Adolescents with Anorexia Nervosa: Preliminary Results
Source: J Clin Med. 2021 Aug 16;10(16):3605. doi: 10.3390/jcm10163605 (PMC8397224; doi:10.3390/jcm10163605)
Supplement: Supplementary file 1 [file jcm-10-03605-s001.zip › jcm-1317244--supplementary.pdf]

## Supplementary materials

**Table S1.** Comparisons of number and length of hospital admissions between patients with and without clinical scores compatible with a diagnosis of ASD.

| | ASD Measures | | | | | | | | | | | |
|---|---|---|---|---|---|---|---|---|---|---|---|---|
| | **ADOS-2 Total Scores** | | | **AQ Total Scores** | | | **ADOS-2 Diagnostic Scores for ASD** | | | **AQ Diagnostic Scores for ASD** | | |
| Treatments | F | *p*-value | Adjusted R2 | F | p-value | Adjusted R2 | F | *p*-value | Adjusted R2 | F | *p*-value | Adjusted R2 |
| N. of hospitalizations | 0.799 | 0.405 | -0.033 | 0.745 | 0.455 | -0.042 | 0.992 | 0.279 | -0.001 | 0.752 | 0.477 | -0.045 |
| Duration of hospitalizations | 0.282 | 0.652 | -0.128 | 0.332 | 0.560 | -0.118 | 0.281 | 0.656 | -0.128 | 0.233 | 0.781 | -0.138 |
| N. of PEU admissions | 0.842 | 0.299 | -0.026 | 0.465 | 0.750 | -0.092 | 1.025 | 0.216 | 0.004 | 0.513 | 0.643 | -0.083 |

Abbreviations: AQ = Autism Questionnaire; ADOS-2 = Autism Diagnostic Observation Schedule-2; ASD = Autism Spectrum Disorder; PEU = Pediatric Emergency Unit. Note: results controlled for baseline EDI-3 Global Psychological Maladjustment (GMPC) and age.

**Table S2.** ASD total scores and treatment intensity.

| | ASD Measures | | | | | |
|---|---|---|---|---|---|---|
| | **ADOS-2 Total** | | | **AQ Total** | | |
| | **OR** | **C.I.** | ***p*-Value** | **OR** | **C.I.** | ***p*-Value** |
| Risperidone | 0.963 | -0.317–0.241 | 0.791 | 0.801 | -0.508–0.064 | 0.128 |
| Aripiprazole | 1.239 | -0.048–0.477 | 0.109 | 1.084 | -0.077–0.238 | 0.315 |
| Olanzapine | 0.920 | -0.513–0.347 | 0.706 | 0.795 | -0.550–0.091 | 0.795 |
| Sertraline | 0.920 | -0.315–0.149 | 0.482 | 0.927 | -0.254–0.101 | 0.401 |
| Fluvoxamine | 0.836 | -0.239–0.194 | 0.836 | 0.998 | -0.147–0.142 | 0.976 |
| Fluoxetine | 1.020 | -0.271–0.310 | 0.894 | 1.425 | -0.040–0.748 | 0.078 |

Abbreviations: AQ = Autism Questionnaire; ADOS-2 = Autism Diagnostic Observation Schedule-2; ASD = Autism Spectrum Disorder. Note: results controlled for baseline EDI-3 Global Psychological Maladjustment (GMPC) and age.
